# Characterization of Circulating Maternal Progestagens, Estrogens, Androgens and Glucocorticoids During Normal Pregnancy in Belugas (*Delphinapterus leucas*) Under Human Care

**DOI:** 10.3390/ani16162550

**Published:** 2026-08-15

**Authors:** Karen J. Steinman, Gisele A. Montano, Todd R. Robeck

**Affiliations:** 1 SeaWorld & Busch Gardens Species Preservation Laboratory, United Parks and Resorts, SeaWorld California, San Diego, CA 92109, USA; gisele.montano@unitedparks.com (G.A.M.);; 2 United Parks and Resorts, Corporate Zoological Operations, SeaWorld Orlando, Orlando, FL 32821, USA

**Keywords:** steroid hormones, pregnancy, beluga, *Delphinapterus leucas*

## Abstract

Determining progesterone concentration is currently considered the most reliable hormonal test for pregnancy detection in belugas. In other cetaceans, additional steroid hormones also serve as pregnancy biomarkers. Whether these hormones are useful for beluga pregnancy diagnosis is unknown. The objective of this study was to conduct circulating steroid hormone analysis across normal pregnancy and different reproductive states in the beluga. Serum samples (n = 240) collected from nine females representative of 20 pregnancies (1991–2017) were analyzed using immunoassays for progesterone, progestagens, estradiol, testosterone, androstenedione, and cortisol. Across pregnancy trimesters (early, mid, late), progesterone was highest during early and mid, and decreased slightly during late, but still maintained concentrations above pre- and post-pregnancy levels. Progestagens increased above luteal phase concentrations during early and continued to increase and remained elevated during mid and late. Estradiol and androstenedione increased during mid and late trimesters. Testosterone was higher at all stages of pregnancy (highest at mid and late) compared to other reproductive states. Across gestation, cortisol was only elevated during late pregnancy. The present study established pregnancy profiles for various steroid hormones during normal beluga pregnancy for animals under human care and identified other non-progestagen hormones that may possibly serve as gestational biomarkers in this species.

## Simple Summary

Pregnancy diagnosis using a single blood sample and testing for a single hormone can be inconclusive in animals, especially in wild populations. This study was the first to examine multiple hormones across several normal pregnancies in belugas under human care. We identified which hormones were the most useful for pregnancy confirmation in our population and at what trimester of pregnancy these hormones are most abundant. Maximizing hormone testing on samples may provide a better understanding of reproductive events and pregnancy dynamics in belugas that can be applied to animals under human care.

## 1. Introduction

The International Union of Conservation and Nature’s Red List classifies the beluga (*Delphinapterus leucas*) as a species of least concern, a status updated in 2017 from its 2008 designation of Near Threatened [1]. However, the Cook Inlet population in Alaska remains listed as Critically Endangered due to significant stock depletion. This status is enforced under the United States Marine Mammal Protection Act [2] and the United States Endangered Species Act [3]. Together, they have catalyzed crucial research and conservation efforts aimed at studying and preserving this vulnerable population.

Effective management and policy implementation for protecting any species requires a foundational understanding of the species’ biology and physiology. Acquiring this knowledge from in situ settings can be difficult, particularly in reproductive physiology, where discrete and subtle changes in estrous cycle dynamics and reproductive failures or losses may be missed due to infrequent sample collection. Ex situ breeding programs in zoological facilities provide an opportunity for in-depth studies into the unique reproductive biology of belugas. Additionally, these populations provide opportunities to validate research methodologies and provide proofs of concept for potential extrapolation of these efforts to in situ populations [4]. Key knowledge areas gained from ex situ studies of belugas include the description of growth and maturation from birth to adulthood [5], the characterization of their estrous cycle, and seasonal reproduction [6].

Progesterone (P4) or the broader class of progestagens remain the primary means for pregnancy identification in both in situ and ex situ cetaceans. Although serum P4 analysis, paired with ultrasonography, is the clinical standard for pregnancy diagnosis in cetaceans under human care [7], its application in wild populations via blubber or serum samples has also shown promise for identifying pregnancy [8,9]. However, emerging research using liquid chromatography tandem mass spectrometry (LC-MS/MS) suggests that P4 alone may be an incomplete diagnostic tool. In belugas and other cetaceans, a diversity and abundance of progestagens and androgens that shift significantly across pregnancy have been identified [10]. Therefore, relying exclusively on P4 for pregnancy diagnosis may lead to missed early-stage pregnancies or an inability to distinguish between normal and potentially abnormal gestations [8,11]. The limitation is exemplified in the bottlenose dolphin (*Tursiops truncatus*), where P4 exhibits a bimodal wave, while progestagens increase linearly across gestation [7]. As a result, relying solely on a single P4 value can obscure accurate gestational stage determination depending on the timing of sample collection.

Other steroid hormones have been analyzed throughout gestation in other cetaceans but have yet to be described in the beluga. Circulating maternal estradiol and cortisol are highest during late gestation in both killer whales [12] and bottlenose dolphins [13]. When using hormone target-specific antibodies in enzyme immunoassays (EIAs), longitudinal increases across gestation have also been observed for androgens, in particular androstenedione (A4) and testosterone (T), in both killer whales [12] and bottlenose dolphins [13].

Pregnancy diagnosis accuracy from single-sample analysis can be improved by utilizing a suite of hormone tests, including progestagens, estrogens, glucocorticoids, and androgens. Beyond diagnosis, this multi-hormone approach may offer insights into reproductive failure [11]. Characterization of hormone profiles during successful gestations (defined as those resulting in a calf surviving beyond 30 days) may have implications for monitoring in situ populations. Subsequently, this retrospective study aimed to describe steroid hormones profiles throughout normal pregnancy in female belugas under human care. Specifically, we sought to: (1) identify the gestational stages at which hormone concentrations deviated from the luteal phase and (2) characterize maternal circulating concentrations of progesterone, progestagens, estradiol, androstenedione, testosterone, and cortisol.

## 2. Materials and Methods

All samples had been previously collected as part of routine veterinary procedures for belugas and at the discretion of the attending veterinarian. The use of these banked samples for this research was reviewed and approved by the United Parks and Resorts Incorporated Research Review Committee. All historical sample collections were performed in strict accordance with the US Animal Welfare Act for the care of marine mammals.

### 2.1. Animals and Time Period

Nine female belugas were included in the study (Table S1), representing 20 normal pregnancies that occurred between 1991 and 2017. Normal pregnancy or gestation was defined as a calf that was born alive and survived for longer than 30 days [7,13]. Belugas were housed at three separate SeaWorld locations in the USA (Orlando, San Antonio, and San Diego, parent company: United Parks and Resorts). Enclosures ranged from a minimum of 258 to 1741 m^3^ of either natural processed or manufactured salt water kept at approximately 17 to 20 °C year-round. Animals were fed a diet consisting primarily of frozen-thawed Pacific herring (*Clupea harengusa*) and/or Columbia River smelt (*Thaleichthys pacificus*) at approximately 4% to 5% of their body weight per day. All fish were graded for human consumption.

### 2.2. Sample Collection

Blood samples were collected monthly during estrous cycles and pregnancy and as part of routine husbandry management (n = 240). Samples were collected unrestrained, with each animal presenting its ventral fluke surface to the attending veterinarian for venipuncture of the central fluke vein using a 19-gauge, 1.5-inch needle. Approximately 20 cc of whole blood was collected into BD Vacutainers (Becton Dickinson, Franklin Lakes, NJ, USA) containing activated thrombin. Samples were centrifuged at 1500 rpm for 10 min, and the serum was decanted and frozen at −80 °C until analysis.

### 2.3. Sample Processing and Extraction

Extraction of beluga sera for use in the androstenedione, testosterone, and cortisol assays was performed identically to previously reported methods used for bottlenose dolphins [13]. Samples were extracted in batches of 25 samples or less (27 total extraction batches). For each sample extraction batch, a pool of beluga sera was spiked with hormone and used as an extraction control to examine the hormone recovery for each batch. Any extraction batch displaying a control recovery of <85% was repeated to ensure adequate efficiency of the procedure. The percentage mean ± sem recovery for the extraction control for each hormone was 96.5 ± 1.2% (n = 27).

### 2.4. Steroid Immunoassays

All steroid concentrations were expressed as ng hormone per ml serum except for estradiol (pg/mL). All samples were analyzed in duplicate for each hormone assay. Any sample with a coefficient of variation (CV) > 10% between replicates was repeated.

#### 2.4.1. Progesterone (P4) Immunoassays

Progesterone was analyzed using an immunofluorescence assay (IFA) at a commercial laboratory, as previously validated for belugas (Quest Diagnostics, Irving, TX, USA) [5,14] or an immunoenzymometric assay (TOSOH Bioscience NV, Tessenderlo, Belgium) as previously described [15]. Assay validations for this species, including sample parallelism and accuracy information, are described in detail in the aforementioned studies. Initially, an outside clinical diagnostic laboratory (Quest Diagnostics) provided progesterone analysis before the in-house laboratories across the three parks uniformly performed the analysis using the immunoenzymometric assay. In a validation trial (as previously described in bottlenose dolphins [15]) comparing paired samples on both assays, concentrations were highly correlated (*r* = 0.853, *p* < 0.05, n = 40), and the limit of detection for both assays was <0.1 ng/mL. Three internal quality controls (Immunoassay Liquichek, BioRad, Hercules, CA, USA) were tested during each run/sample batch and were required to be within the manufacturer’s recommended parameters and concentration ranges for P4 for each batch to be accepted.

#### 2.4.2. Progestagen (Pg) EIA

Progestagen (Pg) concentrations for beluga sera (0.5 to 10 µL) were measured in duplicate using a single-antibody, direct EIA as previously described in detail for use with killer whale sera [16]. Parallel displacement of a pool of beluga sera compared to the standard curve was demonstrated (*r* = 0.994), and an accuracy test plotting the concentrations of a pool of sera spiked with known concentrations of standard against non-spiked sera yielded a recovery of 94.1 ± 11.1% (linear regression, *y* = 1.09*x* + 1.03, *r*^2^ = 0.984, Figure S1). Intra-assay precisions for this and all subsequent assays were determined by analyzing a single sample distributed randomly across the microtiter plate; for the Pg EIA, the intra-assay coefficient of variation (CV) was 8% (n = 20). Inter-assay CVs for the two quality controls at 30% and 70% binding were 11% and 10.5%, respectively (n = 33 assays). The inter-assay CV for a species-specific biological control (S-SBC) made from pooled beluga sera at approximately 50% binding was 11.5% (n = 31 assays).

#### 2.4.3. Estradiol (E2) EIA

Estradiol (E2) concentrations for beluga sera (50 µL) were measured in duplicate using a single-antibody, direct EIA as previously validated for use with bottlenose dolphin sera [13]. Parallel displacement of a pool of beluga sera compared to the standard curve was demonstrated (*r* = 0.954), and an accuracy test plotting the concentrations of a pool of sera spiked with known concentrations of standard against non-spiked sera yielded a recovery of 86.1 ± 3.4% (linear regression, *y* = 0.85*x* + 0.22, *r*^2^ = 0.997, Figure S2). Intra-assay precision was 9.6% (n = 15), while the inter-assay CVs at 30% and 70% binding were 9.8% and 11.6%, respectively (n = 25 assays). The inter-assay CV for an S-SBC at approximately 50% binding was 8% (n = 25 assays).

#### 2.4.4. Androstenedione (A4) Assay

Androstenedione concentrations were measured using a commercial, single-antibody, direct EIA kit (40-056-205044, GenWay Biotech, Inc., San Diego, CA, USA) as previously described for use in the killer whale and bottlenose dolphin [12,13]. Aliquots (5 to 25 µL) of extracted beluga sera were analyzed according to the kit instructions. Cross-reactivity and sensitivity information can be found in the aforementioned publications and manufacturer’s product insert. Parallel displacement of extracted beluga sera compared to the standard curve was demonstrated (*r* = 0.998), and the recovery of known concentrations of standard to extracted serum was 111.4 ± 8.2% (linear regression, *y* = 1.19*x* − 0.6, *r*^2^ = 0.999, Figure S3). Intra-assay precision was 4% (n = 24), while the inter-assay CVs at 20% and 60% binding were 8.5% and 9.2%, respectively (n = 23 assays). The inter-assay CV for an S-SBC at 30% binding was 6.8% (n = 18 assays).

#### 2.4.5. Testosterone (T) Assay

Testosterone concentrations in extracted beluga sera (12.5 to 25 µL) were measured using a single-antibody, direct EIA as previously described in detail for use with killer whale [12]. Cross-reactivity and sensitivity information can be found in the aforementioned publications. Parallel displacement of extracted beluga sera compared to the standard curve was demonstrated (*r* = 0.991), and the recovery of known concentrations of standard to extracted serum was 109 ± 6.6% (linear regression, *y* = 1.1*x* − 0.61, *r*^2^ = 0.994, Figure S4). Intra-assay precision was 9.7% (n = 12), while the inter-assay CVs at 30% and 70% binding were 8.1% and 14.2%, respectively (n = 26 assays). The inter-assay CV for an S-SBC at approximately 60% binding was 11.8% (n = 23 assays).

#### 2.4.6. Cortisol (Cort) Assay

Cortisol concentrations for extracted beluga sera (1 to 25 µL) were measured using a single-antibody, direct EIA as previously described in detail for use with killer whale [12]. Cross reactivity and sensitivity information can be found in the aforementioned publication. Parallel displacement of extracted beluga sera compared to the standard curve was demonstrated (*r* = 0.996), and the recovery of known concentrations of standard to extracted serum was 103.3 ± 10.9% (linear regression, *y* = 1.04*x* − 0.95, *r*^2^ = 0.997, Figure S5). The CV for intra-assay precision was 5.3% (n = 23), while the inter-assay CVs at 30% and 70% binding were 5.3% and 5.1%, respectively (n = 24 assays). The inter-assay CV for an S-SBC at 60% binding was 11.6% (n = 19 assays).

### 2.5. Data and Statistical Analyses

Unless stated otherwise, statistical analyses were performed using Stata statistical software (version 19; StataCorp LP, College Station, TX, USA). To compare hormone concentrations during either different reproductive states or months pre- and post-conception (MPC), and the luteal phase, we employed a mixed-effects restricted maximum likelihood (REML) regression model [12,17]. During model development, the random intercept structure was determined using a likelihood ratio test (LR test) to evaluate whether a two-level (animal ID) or a three-level (pregnancy nested within animal ID) mixed-effects model yielded the best fit (Tables S2–S5). We then compared heteroscedastic residuals (residuals varying by trimester) versus autoregressive residuals (AR1) using AIC to determine the best model fit (Tables S2–S5). The fixed-effect variables were evaluated to compare hormone concentrations (the dependent variable) across reproductive state (or MPC), female age, season, parity, and fetal sex using Kenward-Roger degrees of freedom estimation. Reproductive state was categorized as follows: pre-pregnancy (pre, 1 to 60 days prior to known or estimated conception); first trimester (early, day 1 through 158 post ovulation); second trimester (mid, day 159 through day 314); third trimester (late, day 315 until parturition); post-pregnancy (post, 1 to 60 days post-parturition); and luteal phase (luteal, 7 to 16 days after a non-conceptive ovulation). Season was defined as winter (December–February), spring (March–May), summer (June–August), and fall (September–November). Animal age, parity, and season were included as covariates to control for their effect on the hormone concentrations being evaluated [12].

All final mixed-effects models were checked for normality using quantile–quantile (Q-Q) plots of the standardized residuals. If residuals exhibited a non-normal distribution, data were log- or square root-transformed, guided by the Shapiro–Wilk test (ladder command, Stata), until residuals were normalized. Multiple comparisons of marginal means were performed using Šidák corrections at *p* < 0.05. For text, tables and graphs, transformed data were back-transformed; all results were presented as marginal means with 95% confidence intervals (CI) unless otherwise noted.

## 3. Results

### 3.1. Pregnancy Demographics

We analyzed a total of 20 beluga pregnancies and eight luteal phases from nine individuals that occurred from 1991 through 2017 (Table 1, Table S1). This did not represent the total number of beluga pregnancies that occurred during the study period; rather, it included only those pregnancies with available archived samples of sufficient volume for hormone analysis that met our criteria for a normal pregnancy. For conceptions, 17 (85%) occurred during the spring months (March, April, and May), with the remaining occurring in June. Most births occurred in the summer (June, July, August; n = 17, 85%) and the remainder occurred in September (resulting from June conceptions). Mean pregnancy length was 15.5 months with births occurring during MPCs 14–16.

### 3.2. Steroid Hormones

#### 3.2.1. Random-Effects Model Development

Models for all hormone analyses, whether using state or MPC as the primary time-component fixed-effects variable, were found to provide the best fits using a two-level model (animal ID) with heteroscedastic residuals (Tables S2 and S4).

#### 3.2.2. Progesterone (P4)

Progesterone data required natural log transformation to achieve the best fit based on standardized residual analysis; all data were subsequently log-transformed for statistical analysis, and back-transformed for presentation. For comparisons across independent factors, reproductive state (including pre- and post-pregnancy, luteal phase, and trimesters) was significant (*p* < 0.0001). In addition, omnibus tests indicated both parity (*p* = 0.01) and season (*p* = 0.012) were significant. Progesterone concentrations during all trimesters were significantly higher compared to pre- and post-pregnancy concentrations, while luteal phase concentrations were lower than those observed in early and mid-pregnancy (*p* < 0.05, Table 2). For season, summer (4.6 ng/mL) was greater (*p* < 0.05) than fall (2.3 ng/mL). Primiparous animals (6.2 ng/mL) had the highest concentrations that decreased significantly by calf 3 (2.7 ng/mL, *p* < 0.05).

**Figure 1:**
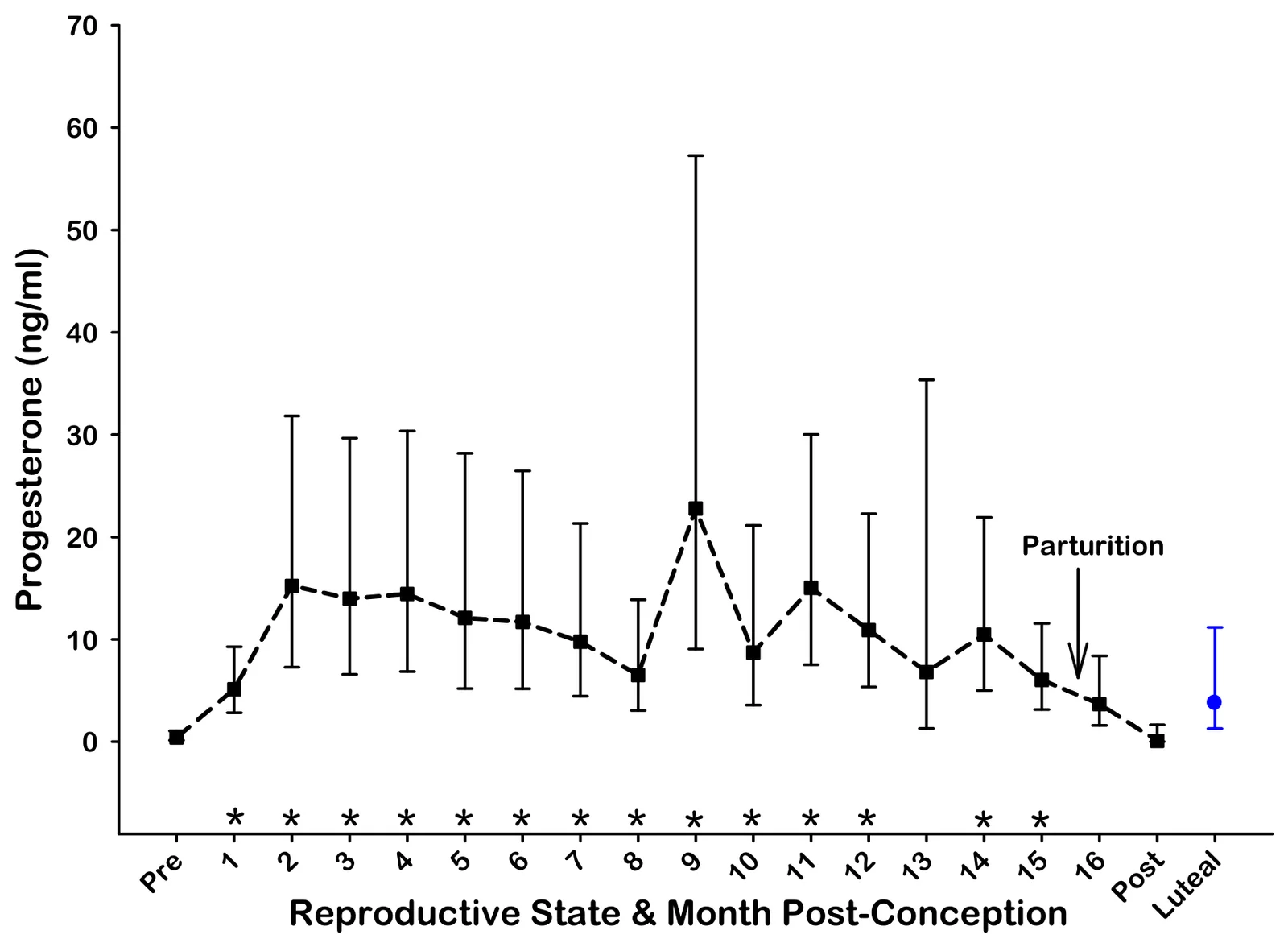
Marginal mean progesterone (P4) concentrations across beluga pregnancy by reproductive state and month post-conception (MPC). The black squares and blue circle represent the back-transformed marginal means for each MPC and luteal phase, respectively. The error bars represent the lower and upper 95% confidence intervals for each marginal mean. Mean parturition date is indicated by an arrow. The black asterisks along the x-axis indicate MPCs with significantly higher P4 concentrations relative to pre-conception (*p* < 0.05).

For MPC, P4 concentrations were greater than (*p* < 0.05) pre-pregnancy concentrations by MPC 1, peaking at MPC 9 (which was significantly higher compared to post-pregnancy) followed by a slow non-significant decline toward parturition. Interestingly, no months post-conception were significantly greater than luteal phase concentrations (Figure 1).

#### 3.2.3. Progestagens (Pg)

Square root transformations of Pg data provided the best fit according to standardized residual analysis (Table S2); therefore, all Pg data were square root-transformed for statistical analysis and back-transformed for data presentation. For comparisons across independent factors, only reproductive state (including pre- and post-pregnancy, luteal phase, and trimesters) was a significant main effect (*p* < 0.0001). Progestagen concentrations across all trimesters of pregnancy were significantly higher than those observed during non-pregnant states (*p* < 0.05, Table 2). Concentrations increased progressively throughout gestation, with mid- and late-gestation levels exceeding early, pre- and post-pregnancy, and luteal phase concentrations (*p* < 0.05, Table 2).

**Figure 2:**
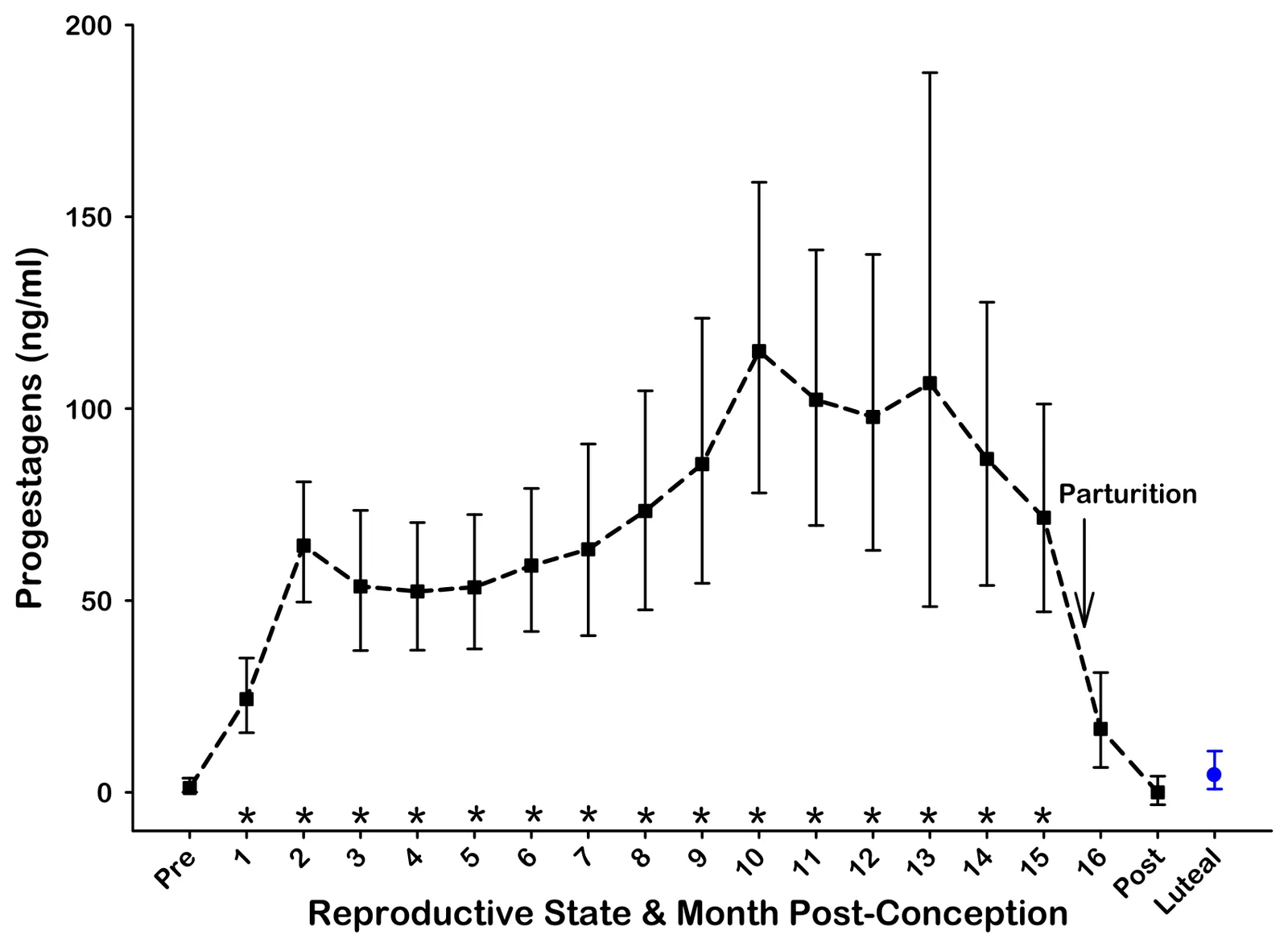
Marginal mean progestagen (Pg) concentrations across beluga pregnancy by reproductive state and month post-conception (MPC). The black squares and blue circle represent the back-transformed marginal means for each MPC and luteal phase, respectively. The error bars represent the lower and upper 95% confidence intervals for each marginal mean. Mean parturition date is indicated by an arrow. The black asterisks along the x-axis indicate MPCs with significantly higher Pg concentrations relative to pre-conception (*p* < 0.05).

Progestagens increased relatively linearly from pre-pregnancy (conception) to MPC 10, then declined through the remainder of gestation (MPC 11–15, parturition, Figure 2). Post-parturition concentrations (MPC 16 and 17), did not differ from pre-gestation levels (*p* > 0.05, Figure 2, Table S3). Concentrations during MPCs 8–12 and 14–15 were higher than those during non-pregnancy (pre, post) and the first and last months of gestation (MPC 1 and 16, *p* < 0.05, Figure 2).

#### 3.2.4. Estradiol (E2)

Estradiol data required natural log transformation to achieve the best fit based on standardized residual analysis (Tables S2 and S3); all data were subsequently log-transformed for statistical analysis, and back-transformed for presentation. For comparisons across independent factors, only reproductive state (*p* < 0.0001, including pre- and post-pregnancy, luteal phase, and trimesters) was a significant main effect. Estradiol concentrations increased throughout gestation, with mid- and late-trimesters levels exceeding early pregnancy and pre- and post-pregnancy levels (*p* < 0.05, Table 2).

**Figure 3:**
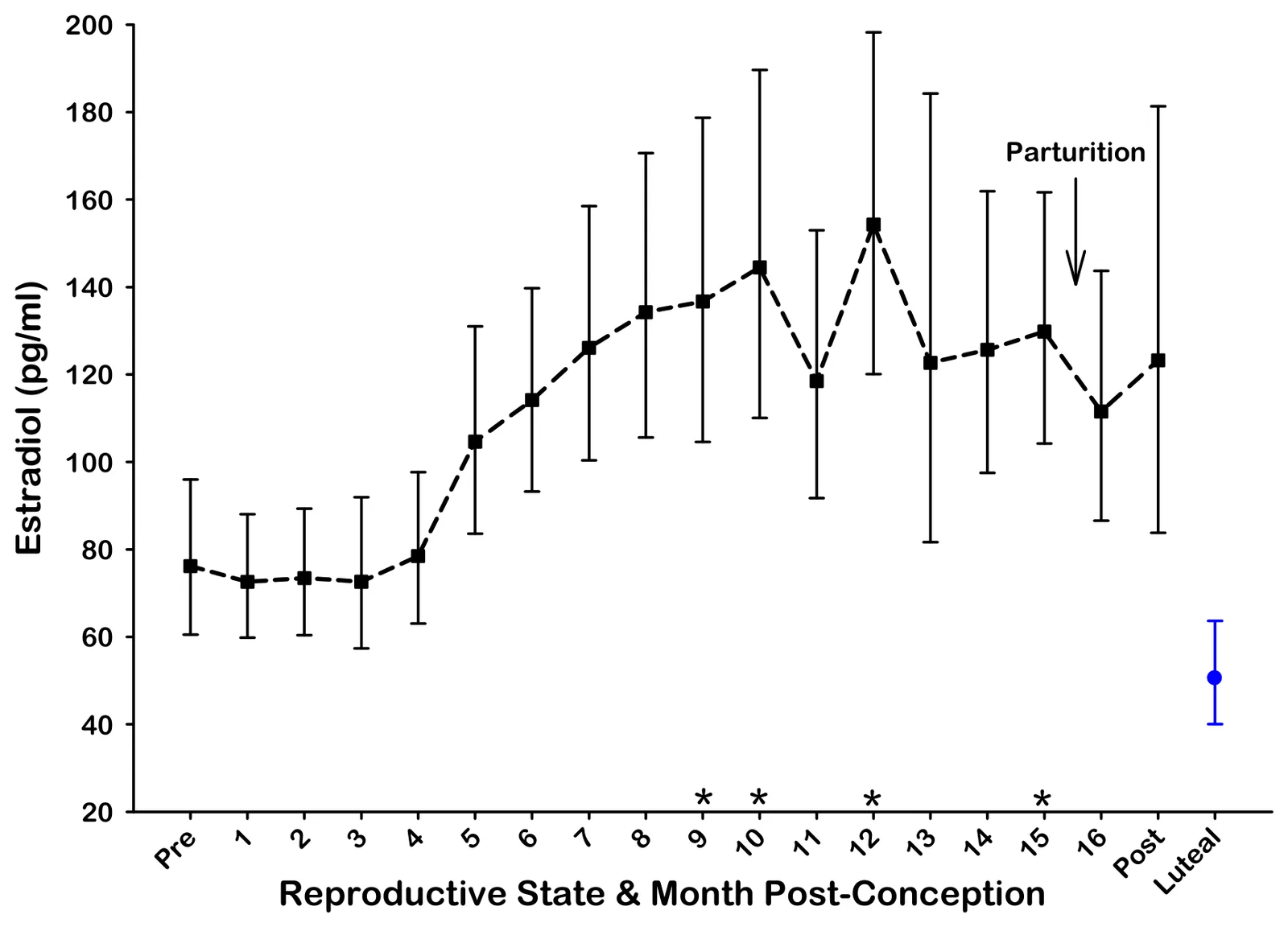
Marginal mean estradiol (E2) concentrations across beluga pregnancy by reproductive state and by month post-conception (MPC). Black lines represent upper and lower 95% confidence intervals. The black squares and blue circle represent the back-transformed marginal mean for each MPC and luteal phase, respectively. The error bars represent the lower and upper 95% confidence intervals for each marginal mean. Mean parturition date is indicated by an arrow. The black asterisks along the x-axis indicate MPCs with significantly higher E2 concentrations relative to pre-conception (*p* < 0.05).

For comparisons across independent factors, MPC (*p* < 0.0001), fetal sex (*p* = 0.03) and parity (*p* = 0.0002) were significant main effects. Peak concentrations occurring at MPC 8, 10, 12 were higher than those during MPCs 1 through 4 (*p* < 0.05, Figure 3). Following this peak, concentrations decreased at MPC 13 and 14, exhibiting a secondary increase at MPC 15 that was also significantly greater than MPCs 1–4 (*p* < 0.05, Figure 3). For fetal sex, animals carrying male fetuses had increased E2 concentrations (106 pg/mL, *p* < 0.05) compared to animals carrying female fetuses (91 pg/mL). Although multiparous females had significantly decreased E2 during their fourth pregnancy (69 pg/mL, *p* < 0.05) compared to other pregnancies (1 = 99, 2 = 111, 3 = 102, 5 = 108 pg/mL), no overall clear trends across parity were observed.

#### 3.2.5. Androstenedione (A4)

Androstenedione (A4) data required a square root transformation to optimize the model fit based on standardized residual analysis (Table S4); all data were subsequently transformed for statistical analysis and back-transformed for data presentation. For comparisons across independent factors, reproductive state (including pre- and post-pregnancy, luteal phase, and trimesters, *p* < 0.0001, Table 2) and season (*p* < 0.0001) were significant. Within season, spring (31 ng/mL) and summer (30.7 ng/mL) were reduced compared to fall (92.9 ng/mL), while spring was also lower than winter (53.5 ng/mL, *p* < 0.05).

**Figure 4:**
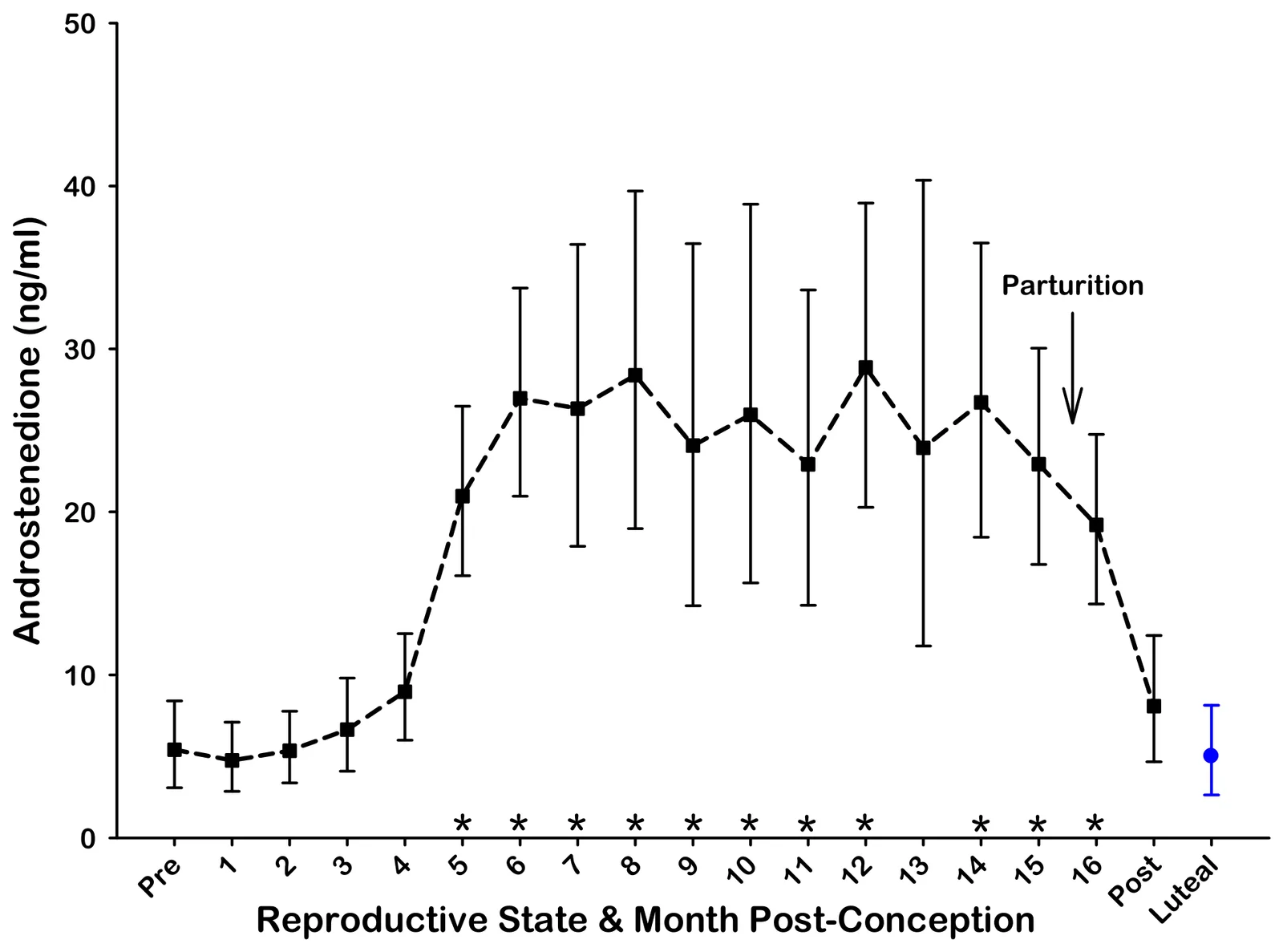
Marginal mean androstenedione (A4) concentrations across beluga pregnancy by reproductive state and by month post-conception (MPC). The black squares and blue circle represent the back-transformed marginal means for each month. The error bars represent the lower and upper 95% confidence intervals for each marginal mean. Mean parturition date is indicated by an arrow. The black asterisks along the x-axis indicate MPCs with significantly higher A4 concentrations relative to pre-conception (*p* < 0.05).

For MPC, concentrations increased throughout pregnancy, with mid- and late-gestation levels significantly elevated compared to early pregnancy, pre- and post-pregnancy, and luteal phases (*p* < 0.05, Table 2). The earliest months of gestation (MPCs 1–4) and non-pregnancy stages (MPCs pre, post, and luteal) exhibited the lowest concentrations and were significantly lower than those during MPCs 8–12 and 14–16 (*p* < 0.05, Figure 4). Marginal mean A4 concentrations during MPCs 5–16 were 2.25- to 6-fold higher than those during MPCs 0–4. Seasonally, concentrations were significant with winter concentrations (102.5 ng/mL) greater than summer (34.1 ng/mL, *p* < 0.05).

#### 3.2.6. Testosterone (T)

Testosterone (T) data required log-transformation to achieve the best fit based on standardized residual analysis (Table S4); all data were subsequently transformed for statistical analysis and back-transformed for data presentation. For comparisons across independent factors, significant fixed effects included reproductive state (including pre- and post-pregnancy, the luteal phase, and trimesters, *p* < 0.0001, Table 2) and season (*p* = 0.0009). Concentrations increased progressively throughout the trimesters of pregnancy, with mid- and late-trimester levels significantly higher than those recorded during early pregnancy. In contrast, pre-pregnancy, post-pregnancy and luteal phase concentrations were significantly lower compared to all other reproductive states (*p* < 0.05, Table 2). For season, spring (0.8 ng/mL) and summer (0.9 ng/mL) were reduced compared to winter (1.4 ng/mL), while spring was lower than fall (1.2 ng/mL, *p* < 0.05).

**Figure 5:**
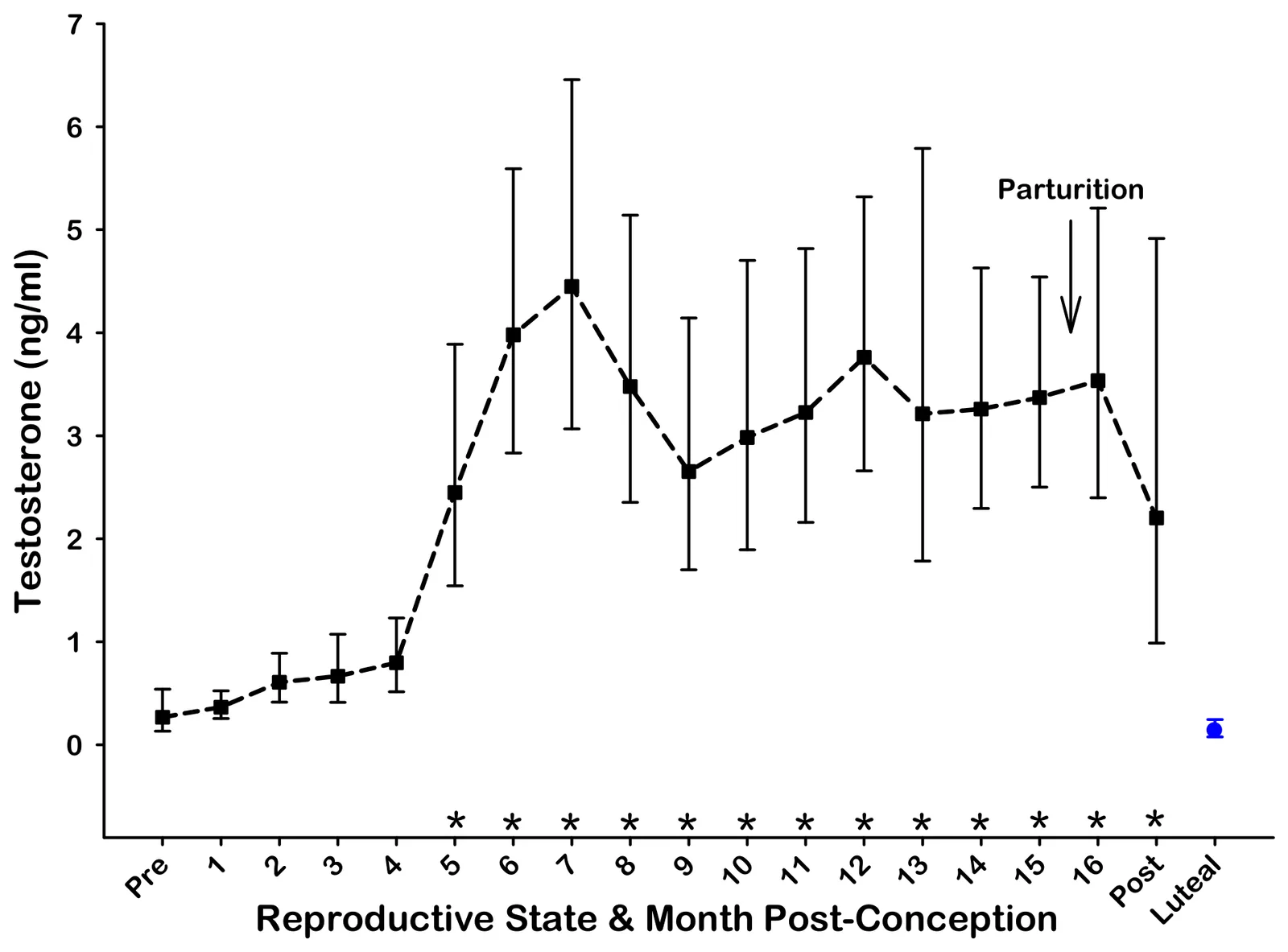
Marginal mean testosterone (T) concentrations across beluga pregnancy by reproductive state and by month post-conception (MPC). The black squares and blue circle represent the back-transformed marginal mean for each month. The error bars represent the lower and upper 95% confidence intervals for each marginal mean. Mean parturition date is indicated by an arrow. The black asterisks along the x-axis indicate MPCs with significantly higher T concentrations relative to pre-conception (*p* < 0.05).

For MPC, significant increases above pre-pregnancy occurred by MPC 5 and continued throughout pregnancy, with maximum concentrations occurring in two phases: during MPCs 6–8 and again from MPC 10 to parturition (*p* < 0.05, Figure 5). Similarly to the analysis with reproductive states, season had a significant effect (*p* = 0.03) with concentrations highest during winter (2.3 ng/mL) compared to fall (1.3 ng/mL, *p* < 0.05).

#### 3.2.7. Cortisol (Cort)

**Figure 6:**
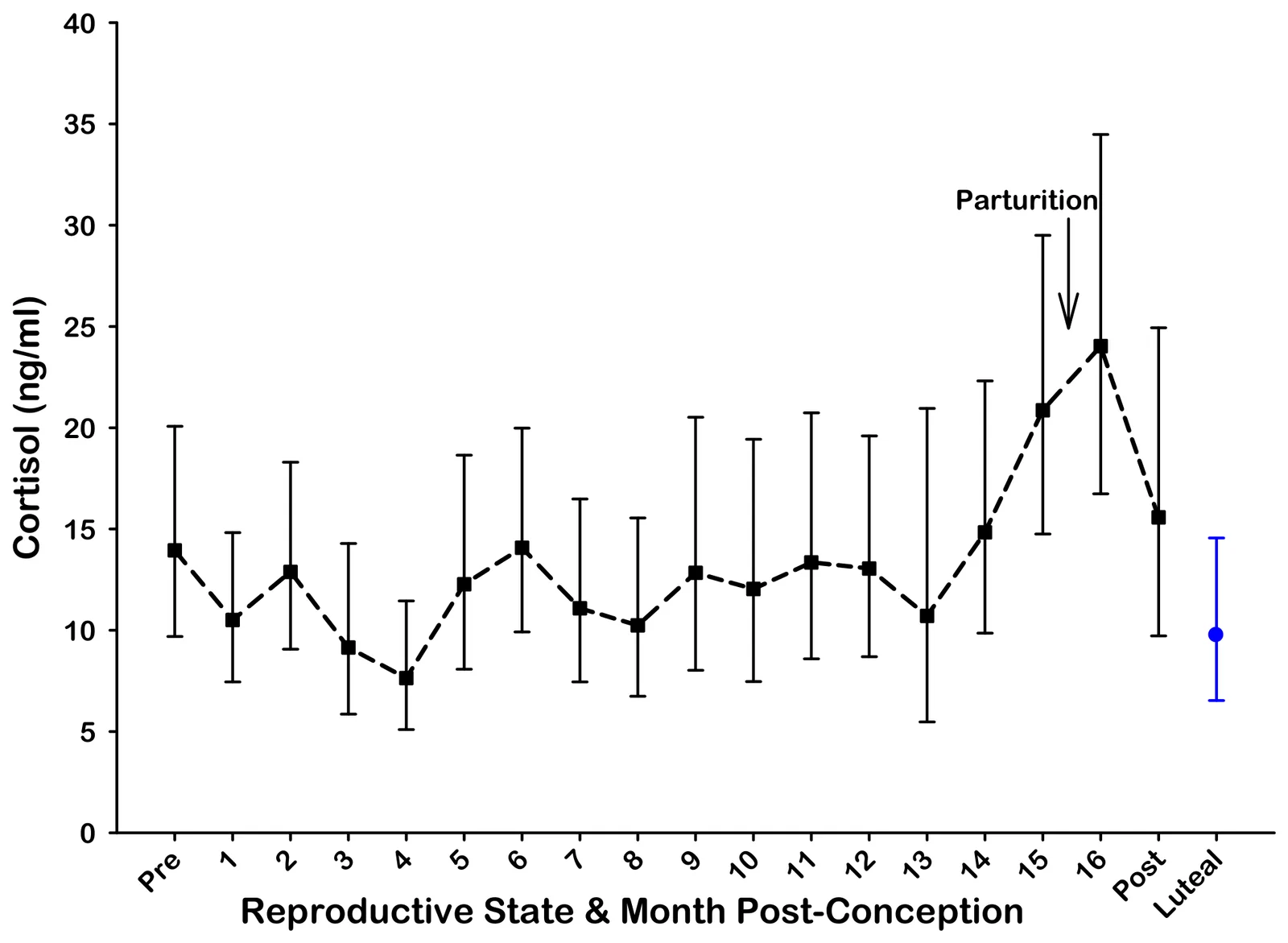
Marginal mean cortisol (Cort) concentrations across beluga pregnancy by reproductive state and by month post-conception (MPC). The black squares and blue circle represent the back-transformed marginal mean for each month. The error bars represent the lower and upper 95% confidence intervals for each marginal mean. The mean parturition date is indicated by an arrow. There were no significant differences in Cort concentrations during pregnancy compared to pre-pregnancy (*p* > 0.05).

Cortisol data required natural log-transformation to achieve the best model fit based on standardized residual analysis (Table S4); all data were subsequently transformed for statistical analysis and back-transformed for data presentation. For comparisons across independent factors, only reproductive state (including pre- and post-pregnancy, luteal phase, and trimesters) was a significant main effect (*p* < 0.0001). Concentrations during late-trimester and post-pregnancy were higher compared to the early trimester (*p* < 0.05, Table 2). For MPC analysis, only MPC 16 had a significant effect (*p* = 0.0003). Although maternally circulating Cort concentrations trended upward across pregnancy, only concentrations at MPC 16 were elevated compared to MPC 3–4 (*p* < 0.05, Figure 6).

## 4. Discussion

We conducted a retrospective analysis of longitudinal steroid hormone profiles throughout normal beluga pregnancy, comparing concentrations across gestational increments (trimesters and months) and other variables including age, season, parity, and reproductive state. As observed in other cetaceans, significant differences in the hormonal milieu were evident across gestational stages. Progesterone (P4) and progestagens (Pg) were most effective for early pregnancy detection, whereas androgens and estradiol were most useful during mid- and late gestation. Cortisol concentrations were significantly elevated only during the final trimester. Implementing a comprehensive hormone panel provides a detailed map of endocrine dynamics that allows for more accurate estimation of conception dates and the characterization of baseline pregnancy profiles during normal pregnancy.

The finding that 85% of conceptions occurred during spring (March through May) reinforces existing reports of seasonal breeding in both in situ [18,19] and ex situ populations [5,20]. To our knowledge, this study provided the first reported parity ranges of belugas. Age at first conception varies from ~4 to 10 years, which is consistent with estimated ages in in situ populations [18,19] and the mean age of 9 years previously reported for belugas under human care [5].

Our mean gestation length of 464 days (range 429 to 485 days, Table 1) aligns with previous ex situ data (467 days) [21] but contrasts with in situ reports ranging from 330 to 441 days [18,19,22]. This discrepancy likely stems from the difficulty of determining definitive conception dates in wild populations. By leveraging frequent blood sampling, ovarian ultrasonography, and daily urine monitoring [5,14] alongside fetal growth measurements [21], we were able to pinpoint conceptions to within a two-week time window, ensuring high diagnostic accuracy for pregnancies lacking an observed breeding date.

### 4.1. Progestagens

The maternal P4 profile in the beluga follows a distinct bimodal wave that is similar to historical observations in individuals under human care [23] and mirrors patterns observed in bottlenose dolphins [15,24,25]. This bimodal pattern has been widely theorized to be indicative of the functional transition from luteal support to placental progestagen production during mid-gestation [16,26]. Our luteal P4 mean (2.9 ng/mL) was within the range of 2.4 to 9.7 ng/mL reported via liquid chromatography tandem mass spectrometry (LC-MS/MS) [10]. Notably, our P4 assay was unable to differentiate pregnancy from the luteal phase at the MPC level unlike what occurs in the bottlenose dolphin [7]. However, at the trimester level, P4 diverged from luteal phase concentrations during early pregnancy. This early divergence at the trimester level is surprising because the relative proportion of P4 within the total pregnane pool remains uniform between the luteal phase and the first trimester. This unique feature of beluga reproductive physiology stands in contrast to the immediate P4 dominance seen in early-trimester bottlenose dolphins and killer whales [10]. This physiologic variance observed in beluga highlights the diagnostic risks associated with relying on a static, single hormone threshold (6 ng/mL) for pregnancy confirmation, as recently suggested by Goertz et al. [8]. For example, applying this rigid benchmark to our longitudinal dataset would have misclassified seven normal gestations due to inconsistently elevated P4 samples throughout pregnancy and natural declines during late pregnancy, while simultaneously misclassifying elevated non-pregnant luteal phase (>15 ng/mL) as false positives [27]. Such diagnostic discrepancies are often exacerbated by inter-study variations of antibody cross-reactivity and statistical modeling frameworks [9,28]. Consequently, a comprehensive monitoring strategy for pregnancies with normal and abnormal outcomes incorporating multi-steroid profiles or alternative biomarkers, like relaxin, is likely essential for accurate assessment of reproductive state, gestational progress, and fetal health [11,29,30].

The potential clinical value of a broader endocrine panel is demonstrated by circulating Pg concentrations, which increased in a progressive linear fashion across the first two trimesters and peaked at MPC 10. This progressive rise captures the metabolic shift toward non-P4 downstream pregnanes, specifically 5α-pregnan-3β,20α-diol [10], detected by the broad cross-reactivity of our Pg antibody with 5α reduced metabolites [7,10]. Although this shift away from upstream P4 dominance is conserved across cetaceans, the quantitative dynamics vary by species. Beluga Pg profiles exhibited a linear accumulation similar to the high-concentration trends in bottlenose dolphins but diverged from the lower-concentration, bimodal fluctuations observed in killer whales [7,16]. Whether these macrolevel differences in progesterone/progestagen production and metabolism during pregnancy are distinctly species-specific or influenced by the lower sample cohorts available for beluga remains to be determined; however, these findings support the theory that the quantitative pathways of progestagen metabolism remain highly specialized.

### 4.2. Estradiol

This study presented the first report of maternal circulating estrogen concentrations during beluga pregnancy. The observation that E2 peaked during mid- and late trimesters compared to early trimesters and the other reproductive physiologic states provides evidence for the placental production of estrogen in belugas. We hypothesize that E2 is primarily of corpus luteum (CL) origin during early pregnancy before shifting to placental production as gestation progresses, a transition well-documented in humans [31] and suspected in other cetaceans [13,16].

In bottlenose dolphins, reduced E2 concentrations are associated with false pregnancies and early embryonic loss [13], reinforcing the theory that the CL is the critical E2 source during early gestation. Disruption in the shift from CL to placental production may negatively impact pregnancy outcomes; therefore, investigating this potential biomarker in cases of abortion or embryonic loss, in comparison to the normal profiles established here, may provide vital diagnostic insights.

The correlation between increased androgen and subsequent E2 elevations suggests a pathway of androgen conversion via aromatization in the fetal liver [32] or placenta [33]. Furthermore, in non-human primates, estrogens are known to activate the fetal hypothalamic–pituitary–adrenal (HPA) axis, stimulating fetal cortisol production [34]. The late gestational cortisol elevations in this study (Table 2, Figure 6) suggest a similar estrogen-induced cortisol mechanism may exist in belugas. Although E2 profiles in belugas mirror other delphinids [13,16,35], the late-pregnancy E2 surge is likely critical for labor initiation [36]. Typically, a rapid P4 decline (removal of the “progesterone block”) in the peri-parturient window is followed by a dramatic E2 increase (see review in López Bernal [37]). Due to the logistical challenges of timing blood collections just prior to labor initiation, we likely missed the definitive pre-parturient P4 drop and the hypothesized periparturient E2 surge.

### 4.3. Androgens

Maternal concentrations of both measured androgens increased significantly as gestation progressed, showing consistency across trimester and MPC levels. This longitudinal increase aligns with data from other cetaceans, as well as prior investigations in belugas [10], North Atlantic right whales (*Eubalaena glacialis*) [38,39], humpback whales [40], bottlenose dolphins [10,13], and killer whales [10,12,41,42]. Although the exact functional role of androgens during cetacean pregnancy remains to be determined, they are likely to serve as precursors for estrogen synthesis. Specifically, the conversion of A4 to estrogens may be a requisite step for the initiation of labor [7]. The specific sources of these androgens, whether maternal, fetal, or placental, require further investigation, especially during pregnancy failure, to better understand their contribution to the endocrine maintenance of pregnancy. Regardless of their source, gestational androgens combined with progestagens appear to provide a robust diagnostic model for determining gestational stage in normal beluga pregnancy [12,41].

In the present study, elevated androgens during gestation compared to non-pregnancy reproductive states (pre-pregnancy, post-pregnancy, and luteal phase) suggest they are pregnancy-specific. Interestingly, T was elevated across all trimesters of pregnancy compared to non-pregnancy states, while A4 differed from non-pregnancy only during mid- and late trimesters, matching patterns observed in both killer whales [12] and bottlenose dolphins [13]. Using LC-MS/MS, Legacki et al. [10] has shown A4 increases in a linear fashion across trimesters during both beluga and killer whale pregnancy. That same study also demonstrates that T, but not 5α-dihydrotestosterone (DHT), is detectable during beluga gestation, albeit at low concentrations that do not change across gestation [10]. Our T assay exhibits high cross-reactivity with DHT and potentially other unknown androgens, which may have led to our increased detectability. Discrepancies in the significance of gestational T concentration patterns when using different analytical methodologies have also been noted in mares [43,44].

When analyzing androgen concentrations at the MPC level, T increased linearly from MPC 4 to 7, followed by sustained elevations through parturition (Figure 5). The overall increasing profile was less pronounced than what has been observed in bottlenose dolphins [13] and killer whales [12]. Concentrations of A4 increased linearly from MPC 4 to 8, then fluctuated until a post-parturition decrease (Figure 4) similar to patterns in killer whales [12]. In contrast, bottlenose dolphins exhibit A4 elevations starting at MPC 4 that peak earlier before a linear decrease until parturition, with the T maximum lagging exactly one month after the A4 peak [13]. Together, these comparative studies demonstrate that the A4 peak preceded the T maximum, and A4 concentrations consistently remain the dominant androgen fraction [12,13]. Downstream testosterone synthesis derived directly from active substrate-driven A4 aromatization could explain these phenomena.

### 4.4. Cortisol

Similar to patterns documented in bottlenose dolphins and killer whales, circulating Cort concentrations in the beluga varied across reproductive states, with concentrations in late pregnancy elevated compared to early pregnancy [12,13]. Most mammals experience a pre-partum increase in glucocorticoids that coincides with late-term organ development and forms a critical component of the peri-parturient hormonal milieu [45]. Increased gestational glucocorticoids or their metabolites have been documented across several cetaceans including killer whales [12,41,42], bottlenose dolphins [18], humpback whales [40], blue whales [46], and North Atlantic right whales [39,47].

Our findings provide further support for glucocorticoid-stimulated fetal development milestones, such as adrenal maturation and surfactant production. In terrestrial mammalian medicine, ante-natal corticosteroid treatment for pregnancies is routinely administered to assist with late-gestation fetal development when premature delivery is likely [48]; this treatment regimen was successfully applied to one beluga with a twin pregnancy [49]. When examined longitudinally by month post-conception, cortisol concentrations remained relatively stable throughout the first two trimesters before rising toward a maternal peak during late gestation, immediately surrounding the parturition window (Figure 6). This observed periparturient concentration increase likely reflects the intense energetic requirements of parturition, nursing and maternal care [50]. Increased sample collection during this period may provide more insights into glucocorticoid influences on parturition and the maternal post-natal adaptations required in caring for a calf.

### 4.5. Seasonal Differences in Steroid Hormones

Interestingly, although a seasonal influence was observed for maternal androgens and progesterone, no significant seasonal effect was detected for E2 concentrations. This contrast may reflect the differing physiologic origins and regulatory mechanisms for these steroid pathways during gestation. Although the progressive, linear increase in Pg concentrations is believed to be driven by placental metabolism, which may shift in response to maternal seasonal metabolic changes, photoperiod, or nutritional intake, E2 production in late pregnancy is typically dictated by a highly conserved, intrinsic fetal-placental clock required to initiate parturition [7]. Consequently, the primary estrogen pathway appears insulated from environmental or seasonal variations that may influence downstream progestagen and androgen concentrations.

Furthermore, because both early and late gestational trimesters frequently overlapped during the spring months in this cohort, a late-pregnancy spike of E2 may mathematically overwhelm more subtle seasonal baselines. Maternal age did not influence estradiol concentrations when evaluated by trimester; however, parity was significant with decreased E2 concentrations observed by the fourth pregnancy. Similarly, in killer whales, multiparous females exhibited lower gestational E2 compared to primiparous individuals, and maternal age also exerts no influence [16]. This suggests that the initial pregnancy event may influence estrogen dynamics in subsequent pregnancies.

Regarding seasonal influences on maternal androgens, T peaked in winter compared to summer and fall, while A4 concentrations were also higher in winter but only compared to summer. Seasonal fluctuations in gestational androgen profiles have been documented in both bottlenose dolphins and killer whales; T is elevated during winter compared to summer for dolphins [13], whereas spring and summer represent the seasons with the highest androgen concentrations in pregnant females [12]. The physiologic mechanism driving these divergent seasonal shifts remains unknown and warrants further investigation.

## 5. Conclusions

This study established the utility of a multi-hormone measurement approach for reproductive analysis in the beluga under human care using target-specific immunoassays. Progestagens and P4 may serve as biomarkers for early pregnancy diagnosis, while androgens, estradiol, and cortisol concentration increases as pregnancy progressed led to differentiation between early, mid- or late gestation in our subjects. These profiles offer vital benchmarks for animal care and husbandry staff in ex situ facilities to monitor gestations longitudinally, particularly when precise conception dates are unknown and diagnostic ultrasonography is unavailable. Extrapolation of these multi-hormone reference ranges via single-sample analysis may be applicable in situ, particularly during the summer months when wild, pregnant females are in either early or late in their gestation. However, our low sample size, repeated pregnancies from the same individuals, sample matrix differences, variability between in situ and ex situ populations and only analyzing normal pregnancy and not abnormal reproductive outcomes limit their diagnostic utility and applicability to in situ settings at present. The capability to accurately determine gestational periods using a suite of biomarkers provides a critical window into population-level reproductive success and can help identify when and where reproductive failures occur. Future investigations targeting pregnancy-specific hormones, such as relaxin, or tracking steroid profiles during gestation with abnormal reproductive outcomes (e.g., spontaneous abortion, early embryonic loss or perinatal mortality) are highly warranted to determine if deviations from normal baselines can effectively differentiate distinct reproductive outcomes.

## Figures and Tables

**Table 1:** Number of females, pregnancies, samples, mean (±SD) age at conception (range), mean (±SD) parity (range), and mean (±SD) pregnancy length (range).

No. of Animals	No. of Pregnancies	No. of Samples	Age (Years)	Parity	Pregnancy Length (Days)
9	20	240	15.7 ± 7.9 (5.2 to 30.8)	1.7 ± 1.4 (0 to 4)	464.3 ± 12.1 (429 to 485)

**Table 2:** Back-transformed marginal mean (95% CI) hormone concentrations (ng/mL unless noted) of progesterone (P4), progestagens (Pg), estradiol (E2), androstenedione (A4), testosterone (T), and cortisol (Cort) during each trimester (early, mid, late) of normal pregnancies, pre- and post-pregnancy, and luteal phase in belugas. Pre: samples collected 1 to 60 days prior to known or estimated conception date; early: day 1 through 158 post-ovulation; mid: day 159 through day 314; late: day 315 until parturition; post: samples collected 1 to 60 days post parturition; luteal: samples collected between 7 and 16 days post ovulation. n = number of samples. If needed, results were transformed for statistical analysis then back-transformed for data presentation (Pg and A4: square root; P4, E2, T, and Cort: log-transformed). Superscripts (^ABCD^) represent significantly different (*p* < 0.05) marginal means among reproductive states for each hormone based on post hoc marginal mean comparisons with Sidak corrections.

Hormone	Pre	Early	Mid	Late	Post	Luteal
P4	0.5 ^A^ (0.3–0.8) n = 7	10.8 ^C^ (7.9–14.8) n = 34	13.8 ^C^ (9.7–19.7) n = 17	8.3 ^BC^ (5.9–11.6) n = 36	0.2 ^A^ (0.1–0.3) n = 10	2.9 ^B^ (1.5–5.5) n = 10
Pg	0.4 ^A^ (0.1–1.1) n = 9	47 ^D^ (40–54.4) n = 59	87 ^C^ (73.1–102.1) n = 36	82.3 ^C^ (68.8–97) n = 53	0.7 ^AB^ (0–2.6) n = 24	3.7 ^B^ (1.9–6.1) n = 15
E2 (pg/mL)	63.5 ^A^ (51.3–78.5) n = 8	85.9 ^A^ (75–98.4) n = 58	117.4 ^BC^ (99.8–138.1) n = 35	145.3 ^C^ (125.3–168.6) n = 52	92.1 ^A^ (69–122.9) n = 24	54.7 ^AB^ (45–66.5) n = 15
A4	6.5 ^A^ (4.7–9) n = 9	9 ^A^ (7–11.3) n = 60	25.2 ^B^ (20.4–30.6) n = 36	29 ^B^ (25–33.3) n = 53	9.2 ^A^ (6.4–12.6) n = 24	5.6 ^A^ (3.8 –7.8) n = 15
T	0.2 ^A^ (0.2–0.3) n = 9	0.9 ^B^ (0.7–1) n = 60	2.9 ^C^ (2.3–3.6) n = 36	4 ^C^ (3.4–4.6) n = 53	0.5 ^A^ (0.3–0.8) n = 24	0.2 ^A^ (0.1–0.3) n = 15
Cort	12.9 ^AB^ (10.3–16.2) n = 9	9.8 ^A^ (8.2–11.7) n = 58	11.8 ^AB^ (9.5–14.5) n = 36	15.3 ^B^ (12.6–18.7) n = 53	18.5 ^B^ (13.3–25.8) n = 24	9.7 ^AB^ (7.6–12.7) n = 15

## Data Availability

The data presented in this study are available on reasonable request from the corresponding author.

## Supplementary Materials

The following supporting information can be downloaded at https://www.mdpi.com/article/10.3390/ani16162550/s1.
