# Supplementary material for: Characterization of Circulating Maternal Progestagens, Estrogens, Androgens and Glucocorticoids During Normal Pregnancy in Belugas (Delphinapterus leucas) Under Human Care
Source: Animals (Basel). 2026 Aug 15;16(16):2550. doi: 10.3390/ani16162550 (PMC13508851; doi:10.3390/ani16162550)
Supplement: Supplementary file 1 [file animals-16-02550-s001.zip › animals-4437410-supplementary.pdf]

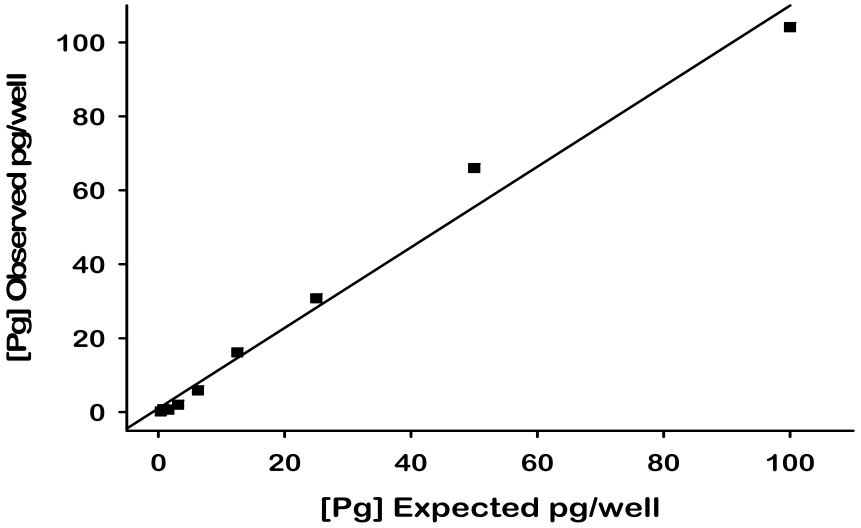

Figure S1. Progestagen (Pg) accuracy test. Concentrations of a pool of beluga sera spiked with known concentrations of Pg against non-spiked sera (black squares). X- and Y-axes represent the expected and observed concentrations, respectively. Recovery was 94.1 ± 11.1%, linear regression (black line), $y = 1.09x + 1.03$, $r^2 = 0.984$.

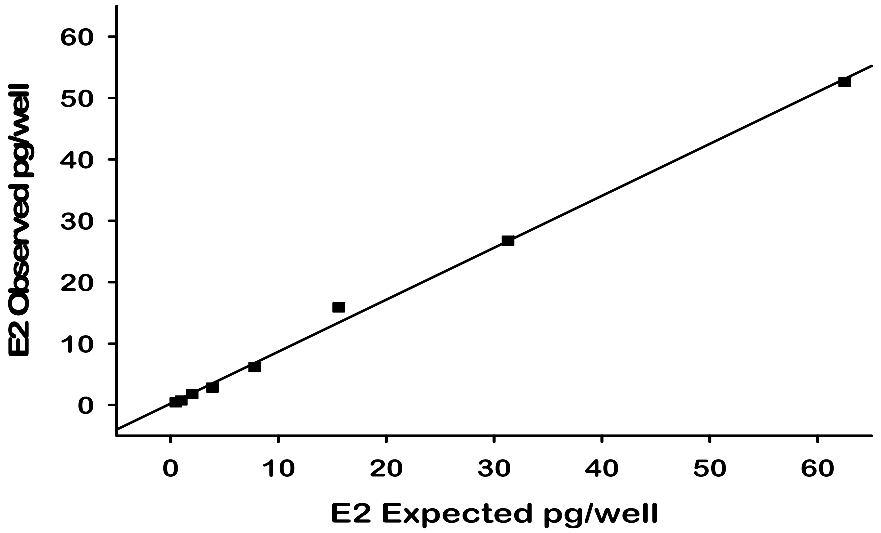

Figure S2. Estradiol (E2) accuracy test. Concentrations of a pool of beluga sera spiked with known concentrations of E2 against non-spiked sera (black squares). X- and Y-axes represent the expected and observed concentrations, respectively. Recovery was 86.1 ± 3.4%, linear regression (black line), $y = 0.85x + 0.22$, $r^2 = 0.997$.

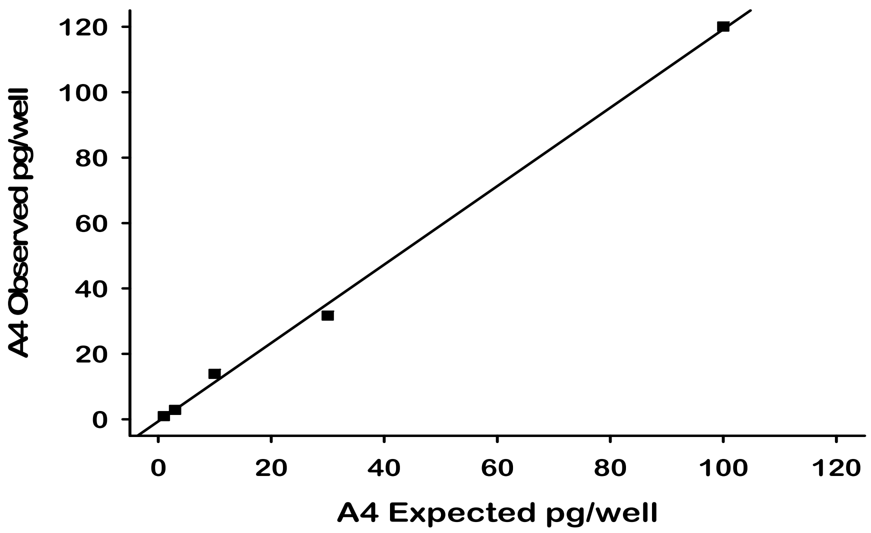

Figure S3. Androstenedione (A4) accuracy test. Concentrations of a pool of extracted beluga sera spiked with known concentrations of A4 against extracted, non-spiked sera (black squares). X- and Y-axes represent the expected and observed concentrations, respectively. Recovery was 111.4 ± 8.2%, linear regression (black line), $y = 1.19x – 0.6$, $r^2 = 0.999$.

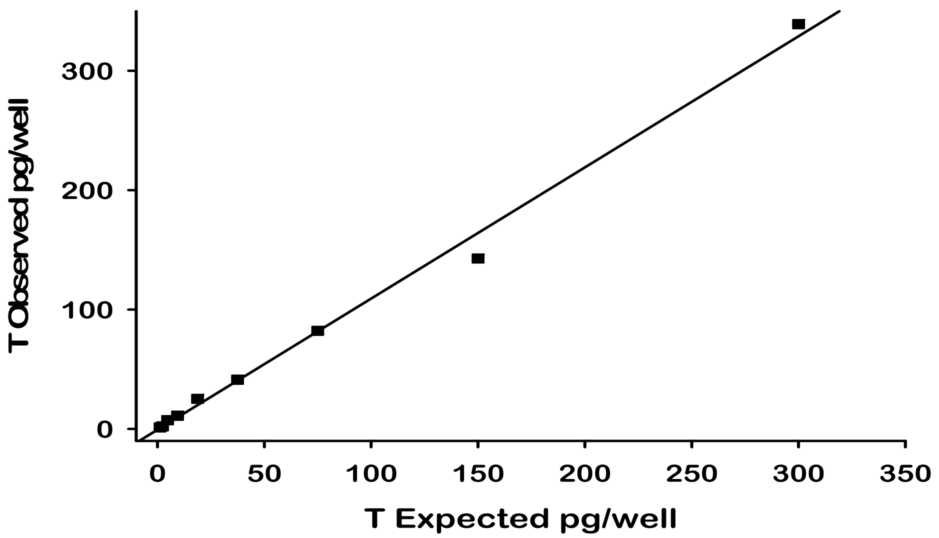

Figure S4. Testosterone (T) accuracy test. Concentrations of a pool of extracted beluga sera spiked with known concentrations of T against extracted, non-spiked sera (black squares). X- and Y-axes represent the expected and observed concentrations, respectively. Recovery was 109 ± 6.6%, linear regression (black line), $y = 1.1x - 0.61$, $r^2 = 0.994$.

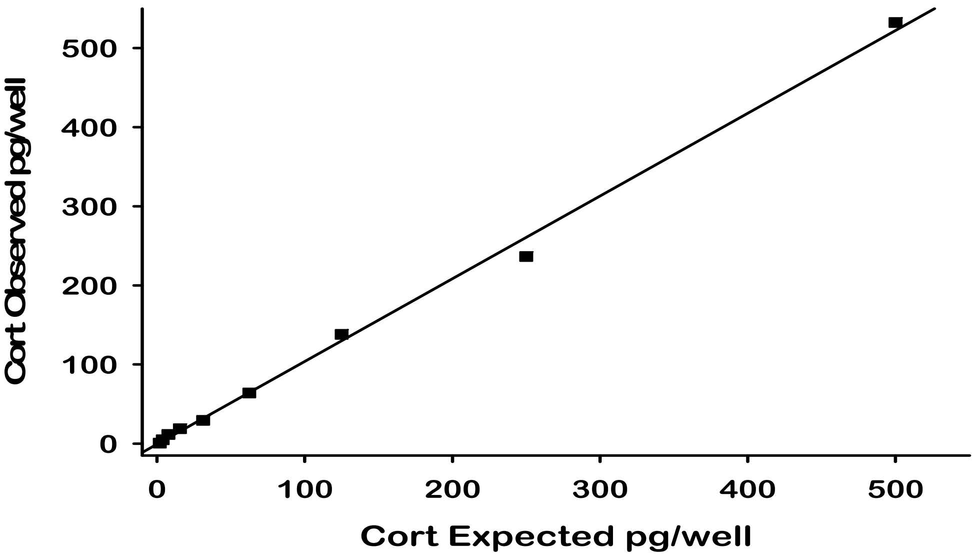

Figure S5. Cortisol (Cort) accuracy test. Concentrations of a pool of extracted beluga sera spiked with known concentrations of Cort against extracted, non-spiked sera (black squares). X- and Y-axes represent the expected and observed concentrations, respectively. Recovery was 103.3 ± 10.9%, linear regression (black line), $y = 1.04x – 0.95$, $r^2 = 0.997$.

Table S1. Study animal demographic information.

Animal #	Date of Birth	Conception Date	Parturition Date
1	13 Feb 1986	30 Apr 1991	8 Aug 1992
		17 Apr 1998	27 Jul 1999
2	1 Jan 1986	11 Apr 1998	25 Jul 1999
		27 Mar 2008	12 Jun 2009
		14 May 2012	26 Jul 2013
		14 Jun 2016	17 Sep 2017
3	1 Jan 1984	1 Jun 1992	25 Sep 1993
4	4 Jul 2000	4 Apr 2009	23 Jun 2010
		1 Apr 2012	9 Jul 2013
		13 May 2015	11 Aug 2016
5	1 Jan 1986	16 May 1994	19 Jul 1995
		18 Mar 2006	26 Jun 2007
6	18 Jul 1986	5 Jun 1992	13 Sep 1993
		6 Mar 1997	19 Jun 1998
		16 Apr 1999	24 Jul 2000
		13 Mar 2006	23 Jun 2007
7	1 Jan 1979	28 Apr 1990	7 Aug 1991
		18 Apr 1993	28 Jul 1994
8	15 Jun 1978	15 Mar 2009	23 Jun 2010
9	1 Jan 1984	26 Apr 2007	31 Jul 2008

Table S2. Random effects model development for progesterone, progestagens, and estradiol. The random intercept structure was determined using a likelihood ratio test (LR test) to evaluate whether a two-level (animal ID) or a three-level (pregnancy nested within animal ID) mixed-effects model yielded the best fit. Then heteroscedastic residuals (residuals varying by trimester or month post-conception [MPC]) versus autoregressive residuals (AR1) were compared using Akaike information criterion (AIC) to determine the best model fit.

**Endocrine Analyte**	**Progesterone**		**Progestagens**		**Estradiol**	
	**Trimester**	**MPC**	**Trimester**	**MPC**	**Trimester**	**MPC**
**Transformation**	Natural log (lnx)	Natural log (lnx)	Square-root (x)	Square-root (x)	Natural log (lnx)	Natural log (lnx)
**Random Heirarchy**	2-Level (id)	2-Level (id)	2-Level (id)	2-Level (id)	2-Level (id)	2-Level (id)
**LR Test p (id)**	0.36	0.52	1.00	1.00	0.09	0.50
**Trimester-Heteroskedastic AIC**	365.0	302.0	1149.1	1099.9	318.7	228.7
**AR1 AIC**	374.8	339.0	1243.4	1153.0	378.8	287.8
**ΔAIC**	-9.8	-37.0	-94.3	-53.1	-55.1	-59.1
**Preferred Residual Structure**	Trimester Heteroskedastic	Trimester Heteroskedastic	Trimester Heteroskedastic	Trimester Heteroskedastic	Trimester Heteroskedastic	Trimester Heteroskedastic

Table S3. Mixed-effects restricted maximum likelihood (REML) regression model. The fixed-effect variables that were evaluated to compare hormone concentrations (the dependent variable) across reproductive state (Trimester [Tri] or month post-conception [MPC]), female age, season, parity, and fetal sex using Kenward-Roger degrees of freedom estimation. Animal age, parity, and season were included as covariates to control for their effect on the hormone concentrations being evaluated.

**Endocrine Analyte**	**Dependent variable**					
	**Progesterone**		**Progestagens**		**Estradiol**	
	**Trimester**	**MPC**	**Trimester**	**MPC**	**Trimester**	**MPC**
**Transformation**	Natural log (lnx)	Natural log (lnx)	Square-root (x)	Square-root (x)	Natural log (lnx)	Natural log (lnx)
**Overall Model F--Statistic**	chi2(15)=633.37	chi2(28)=143.38	chi2(16)=1326.96	chi2(29)=931.59	chi2(16)=245.95	chi2(29)=275.02
**Model P-value**	<0.0001	<0.0001	<0.0001	<0.0001	<0.0001	<0.0001
**LR Test Random vs Linear Regression**	chi2(1)=15.23 (0.0186)	chi2(1)=29.25 (<0.0001)	chi2(1)=107.3 (<0.0001)	chi2(1)=67.66 (<0.0001)	chi2(1)=76.66 (<0.0001)	chi2(1)=69.4 (<0.0001)
**Tri/MPC F-Statistic**	chi2(5)=416.68 (<0.0001)	chi2(18)=97.35 (<0.0001)	chi2(5)=909.64 (<0.0001)	chi2(18)=532.1 (<0.0001)	chi2(5)=124.58 (<0.0001)	chi2(18)=132.46 (<0.0001)
**Season F-Statistic**	chi2(3)=10.88 (0.0124)	chi2(3)=2.07 (0.5581)	chi2(3)=3.13 (0.3723)	chi2(3)=5.28 (0.1521)	chi2(3)=11.11 (0.0111)	chi2(3)=3.15 (0.3689)
**Parity F-Statistic**	chi2(4)=14.87 (0.005)	chi2(4)=7.81 (0.0989)	chi2(5)=1.82 (0.8728)	chi2(5)=3.39 (0.6402)	chi2(5)=20.56 (0.001)	chi2(5)=24.08 (0.0002)
**Fetal sex F-Statistic**	chi2(2)=2.98 (0.2253)	chi2(2)=3.17 (0.2049)	chi2(2)=4.48 (0.1067)	chi2(2)=3.06 (0.2168)	chi2(2)=6.14 (0.0465)	chi2(2)=7.11 (0.0286)
**Maternal age F-Statistic**	chi2(1)=2.86 (0.0908)	chi2(1)=1.1 (0.2947)	chi2(1)=0.12 (0.7319)	chi2(1)=0.01 (0.9289)	chi2(1)=0.33 (0.5678)	chi2(1)=0.31 (0.5789)

Table S4. Random effects model development for androstenedione, testosterone, and cortisol. The random intercept structure was determined using a likelihood ratio test (LR test) to evaluate whether a two-level (animal ID) or a three-level (pregnancy nested within animal ID) mixed-effects model yielded the best fit. Then heteroscedastic residuals (residuals varying by trimester or month post-conception [MPC]) versus autoregressive residuals (AR1) were compared using Akaike information criterion (AIC) to determine the best model fit.

**Endocrine Analyte**	**Androstenedione**		**Testosterone**		**Cortisol**	
	**Trimester**	**MPC**	**Trimester**	**MPC**	**Trimester**	**MPC**
**Transformation**	Square-root (x)	Square-root (x)	Natural log (lnx)	Natural log (lnx)	Natural log (lnx)	Natural log (lnx)
**Random Heirarchy**	2-Level (id)	2-Level (id)	2-Level (id)	2-Level (id)	2-Level (id)	2-Level (id)
**LR Test p (id)**	1.00	1.00	0.49	0.55	0.01	0.43
**Trimester-Heteroskedastic AIC**	860.3	740.0	709.7	590.1	575.99	493.39
**AR1 AIC**	870.3	767.8	741.8	646.2	587.78	500.90
**ΔAIC**	-10.0	-27.7	-32.1	-56.2	-11.79	-7.51
**Preferred Residual Structure**	Trimester Heteroskedastic	Trimester Heteroskedastic	Trimester Heteroskedastic	Trimester Heteroskedastic	Trimester Heteroskedastic	Trimester Heteroskedastic

Table S5. Mixed-effects restricted maximum likelihood (REML) regression model. The fixed-effect variables that were evaluated to compare hormone concentrations (the dependent variable) across reproductive state (Trimester [Tri] or month post-conception [MPC]), female age, season, parity, and fetal sex using Kenward-Roger degrees of freedom estimation. Animal age, parity, and season were included as covariates to control for their effect on the hormone concentrations being evaluated.

	**Dependent variable**					
**Endocrine Analyte**	**Androstenedione**		**Testosterone**		**Cortisol**	
	**Trimester**	**MPC**	**Trimester**	**MPC**	**Trimester**	**MPC**
**Transformation**	Square-root (x)	Square-root (x)	Natural log (lnx)	Natural log (lnx)	Natural log (lnx)	Natural log (lnx)
**Overall Model F--Statistic**	chi2(16)=442.39	chi2(29)=493.09	chi2(16)=675.11	chi2(29)=521.63	chi2(16)=50.49	chi2(29)=76.08
**Model P-value**	<0.0001	<0.0001	<0.0001	<0.0001	<0.0001	<0.0001
**LR Test Random vs Linear Regression**	chi2(1)=37.35 (<0.0001)	chi2(1)=42.51 (<0.0001)	chi2(1)=47.2 (<0.0001)	chi2(1)=59.4 (<0.0001)	chi2(1)=27.27 (0.0001)	chi2(1)=19.86 (0.0029)
**Tri/MPC F-Statistic**	chi2(5)=284.44 (<0.0001)	chi2(18)=249.49 (<0.0001)	chi2(5)=438.75 (<0.0001)	chi2(18)=344.4 (<0.0001)	chi2(5)=27.67 (<0.0001)	chi2(18)=45.59 (0.0003)
**Season F-Statistic**	chi2(3)=28.59 (<0.0001)	chi2(3)=11.15 (0.011)	chi2(3)=16.36 (0.001)	chi2(3)=8.94 (0.0301)	chi2(3)=1.89 (0.5947)	chi2(3)=1.78 (0.6203)
**Parity F-Statistic**	chi2(5)=8.44 (0.1336)	chi2(5)=11.74 (0.0386)	chi2(5)=7.62 (0.1782)	chi2(5)=9.25 (0.0995)	chi2(5)=5.04 (0.411)	chi2(5)=5.51 (0.3574)
**Fetal sex F-Statistic**	chi2(2)=2.3 (0.3164)	chi2(2)=1.52 (0.4666)	chi2(2)=0.3 (0.8616)	chi2(2)=0.42 (0.8103)	chi2(2)=1.31 (0.5183)	chi2(2)=1.65 (0.4386)
**Maternal age F-Statistic**	chi2(1)=0.08 (0.7772)	chi2(1)=0 (0.9589)	chi2(1)=0.46 (0.4967)	chi2(1)=0.01 (0.927)	chi2(1)=0.26 (0.6106)	chi2(1)=0.03 (0.8595)
